# Phylogenetic analysis of modularity in protein interaction networks

**DOI:** 10.1186/1471-2105-10-333

**Published:** 2009-10-14

**Authors:** Sinan Erten, Xin Li, Gurkan Bebek, Jing Li, Mehmet Koyutürk

**Affiliations:** 1 Department of Electrical Engineering & Computer Science, Case Western Reserve University, Cleveland, USA; 2 Case Center for Proteomics & Bioinformatics, Case Western Reserve University, Cleveland, USA; 3 Case Comprehensive Cancer Center, Case Western Reserve University, Cleveland, USA; 4 Department of Epidemiology and Biostatistics, Case Western Reserve University, Cleveland, USA; 5 Genomic Medicine Institute, Cleveland Clinic, Cleveland, USA

## Abstract

**Background:**

In systems biology, comparative analyses of molecular interactions across diverse species indicate that conservation and divergence of networks can be used to understand functional evolution from a systems perspective. A key characteristic of these networks is their modularity, which contributes significantly to their robustness, as well as adaptability. Consequently, analysis of modular network structures from a phylogenetic perspective may be useful in understanding the emergence, conservation, and diversification of functional modularity.

**Results:**

In this paper, we propose a phylogenetic framework for analyzing network modules, with applications that extend well beyond network-based phylogeny reconstruction. Our approach is based on identification of modular network components from each network separately, followed by projection of these modules onto the networks of other species to compare different networks. Subsequently, we use the conservation of various modules in each network to assess the similarity between different networks. Compared to traditional methods that rely on topological comparisons, our approach has key advantages in (*i*) avoiding intractable graph comparison problems in comparative network analysis, (*ii*) accounting for noise and missing data through flexible treatment of network conservation, and (*iii*) providing insights on the evolution of biological systems through investigation of the evolutionary trajectories of network modules. We test our method, MOPHY, on synthetic data generated by simulation of network evolution, as well as existing protein-protein interaction data for seven diverse species. Comprehensive experimental results show that MOPHY is promising in reconstructing evolutionary histories of extant networks based on conservation of modularity, it is highly robust to noise, and outperforms existing methods that quantify network similarity in terms of conservation of network topology.

**Conclusion:**

These results establish modularity and network proximity as useful features in comparative network analysis and motivate detailed studies of the evolutionary histories of network modules.

## Background

As a fundamental concept, evolution has profound implications in a variety of applications in modern molecular biology; *e.g*., functional annotation of DNA/protein sequences through comparative sequence analysis has become an important and integral part of biological sciences [1]. Accurate reconstruction of the evolutionary history of species, usually represented by a phylogenetic tree, is critical for the success of such applications. Phylogenetic analysis of molecular sequence data has drawn significant attention ever since protein/DNA sequences have become available [2,3]. There exist many models (from the simplest Jukes-Cantor model to more complex General Time Reversible model), but all of them specify site evolutions at the DNA level for obvious reasons: structure constraints (secondary structures of RNAs and tertiary structures of proteins) are hard to model. Based on sequence evolution, different approaches have been developed to either explicitly use an evolutionary model (*e.g*., Maximum Likelihood) or approximate one (*e.g*., Maximum Parsimony) [4].

### Understanding functional evolution

It is clear that many other evolutionary constraints (*e.g*., structure, function) are not considered in phylogenetic analyses using conventional approaches based on sequence comparisons. As the genome of an organism is affected throughout evolution, the structure and hence the inner dynamics of networks representing the functional relationships of these genes evolve in parallel with the genome. Most changes on genes, as little as one residue, might affect the functional relationships among interacting biomolecules. These functional relationships include transcriptional and translational regulation, protein-protein interactions, gene modifications, post-translational protein modifications, metabolic reactions, and indirect interactions such as genetic interactions (*e.g*., synthetic lethality). The structure of these networks can vary over time and space, accounting for the dynamics of the system [5]. While networks contain a more solid base for understanding biological systems, the evolutionary path that is taken by cellular organization and cellular signaling is yet to be uncovered.

### Comparative network analysis

Availability of high-throughput data that relates to the organization and dynamics of biological systems enables understanding of biological functions from a systems perspective [6]. An important source of data that pertains cellular organization and signaling is in the form of physical interactions between proteins, organized into genome scale protein-protein interaction (PPI) networks [7]. Comparison of recently available PPI networks that belong to diverse model organisms reveals that parts of extant molecular networks are conserved across diverse species [8-10]. Furthermore, it is observed that proteins that are organized into cohesive interaction patterns are more likely to be conserved [11]. Comparative network analysis is also shown to enhance the performance of computational approaches to basic problems in functional genomics, such as identification of orthologs across species and annotation of protein functions [12,13]. Furthermore, recent studies show that incorporation of evolutionary models and knowledge enhances the performance of network alignment methods significantly [14]. Such findings demonstrate that network comparisons provide essential biological information beyond what is gleaned from the genome [15]. Consequently, phylogenetic analysis of network topology and function has the potential to provide key insights on the evolution of biological functions at a systems level [16].

### Phylogenetic analysis of network modularity

**Figure 1:**
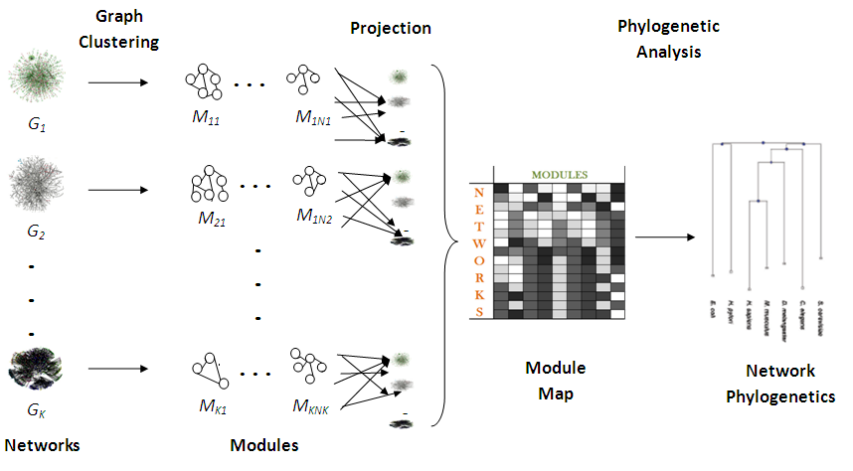
**Modularity Based Phylogenetic Analysis of Molecular Interaction Networks**.

In this paper, we propose a modularity based approach to phylogenetic network analysis. The proposed framework, MOPHY, is illustrated in Figure 1. Our approach differs fundamentally from existing approaches, in that we focus on the conservation and divergence of modular components, rather than one-to-one comparison of network topologies. In our framework, we first identify modular subgraphs in different networks independently. Then, we project these modules on networks of other species to understand the conservation and divergence of different modular processes in these networks. While projecting a module on different species, we rely on the conservation of network proximity between homologs (proteins with significant sequence similarity) of its constituent proteins in other networks. Consequently, by utilizing network information, our approach captures functional evolution beyond conservation of sequences. Namely, network information is incorporated into the analysis by (*i*) considering network modules as "features" of each network and (*ii*) assessing the conservation of modularity in terms of the network proximity between proteins with conserved sequences.

Our approach is motivated by several observations:

• Existing evidence suggests that modularity plays an important role in the evolution of biological systems [17]. Therefore, by explicitly incorporating modularity into the analysis, we can capture the evolutionary relationships between extant networks more accurately.

• Explicit identification and projection of modules also enables the study of the evolutionary histories of specific modules, identification of module families, and construction of module-specific phylogenies [18].

• Available interaction data are highly incomplete and noisy [19]. Compared to topology based analysis, modularity based analysis is likely to be more robust to such imperfections in the data, since it relies on conservation of aggregate network properties (*i.e*., network proximity and modularity), rather than conservation of individual interactions.

• Comparative network analysis is computationally expensive because of the intractability of the underlying subgraph isomorphism problem, difficulties in formulating and accounting for approximate matches, and existence of multiple mappings between proteins in different species [9,10]. Modularity- based analysis alleviates this problem by avoiding one-to-one comparison of network topologies.

### Evaluating network comparison methods

In this study, we also take a novel approach to the calibration and validation of comparative network analysis methods. Based on theoretical models on the evolution of molecular interactions [20,21], we simulate network evolution to generate networks with known underlying phylogeny. Then, we use our algorithms on the generated networks to reconstruct a phylogenetic tree. By comparing the reconstructed tree with the underlying tree, we evaluate the performance of different methods and assess the effect of various parameters on the accuracy of our methods. We also use simulated networks to evaluate the robustness of our methods against noise and missing data, by perturbing the simulated networks to mimic data collection processes. Extensive experiments on simulated data show that the proposed algorithm is extremely successful in reconstructing the underlying phylogenies and is quite robust to noise. We also use the proposed method, MOPHY, to reconstruct the phylogeny of seven species, for which reasonable interaction data is available. We show that our method constructs a phylogenetic tree that is in accordance with the phylogenetic relationships and evolutionary distances inferred by independent methods. Furthermore, we demonstrate that MOPHY outperforms existing phylogenetic network analysis methods.

### Learning from phylogenetic network analysis

It should be noted that the application of the proposed framework extends well beyond phylogenetic tree reconstruction. The methods and results presented here rather constitute a step towards establishing modularity based analysis as a key approach in understanding the functional evolution of cellular organization. Indeed, our results show that conservation of modularity and network proximity is likely to provide useful insights into the evolutionary histories of networks, by providing statistical evidence for the following observations:

• Conservation of network proximity is a better indicator of evolutionary relationships when modular network components are considered (as opposed to the proximity between arbitrary proteins) (Tables 1, 2 and 3).

**Table 1:** Comparison of performances of MOPHY with using random protein modules, using protein similarities only, using random homologous protein partners and RDL.

**METHOD**	**Run 1**	**Run 2**	**Run 3**	**Run 4**	**Run 5**	**Avg**.
MOPHY	5.28	5.85	8.82	5.82	8.15	6.79
RDL	14.40	15.50	19.80	13.12	14.83	15.53
Random Modules	14.81	9.33	11.54	8.11	12.6	11.29
Only Protein Similarity	11.72	11.56	13.96	11.22	10.75	11.84
Random Homolog Selection	15.01	13.60	17.73	22.72	17.61	17.33

For the simulated networks, we compare the performance of MOPHY with the random module method, random homolog selection method, RDL and a method that only uses protein similarity in the networks. Nodal distance for five simulation instances as well as the average values of these five runs are shown in the table. For MOPHY, the result used is the best performance achieved with coverage 0.60 and diameter 2, by using the most specific modules. Similarly for the random module method, the best performance is achieved for the instance with coverage 0.40 and diameter 4, with the most comprehensive modules. For the random homolog selection method, the best result is achieved with coverage 0.60 and diameter 3, by using the most specific modules. As clearly seen, MOPHY outperforms the other methods in terms of capturing the evolutionary distances between species.

**Table 2:** Performance of MOPHY in capturing the topology of underlying phylogeny for simulated networks.

***Most Specific Modules***									
	**Diameter**								
	
	**2**			**3**			**4**		

**Coverage**	**MOPHY**	**Random**	***p*-value**	**MOPHY**	**Random**	***p*-value**	**MOPHY**	**Random**	***p*-value**

20%	1.6**	11.2	0.0039	1.6**	11.2	0.0039	1.6**	12.0	0.0029

40%	1.6**	12.0	0.0013	1.6**	10.8	0.0093	1.6**	12.0	0.0014

60%	1.6**	11.2	0.0019	1.6**	11.6	0.0039	1.6**	11.6	0.0021

***Most Comprehensive Modules***									

	**Diameter**								
	
	**2**			**3**			**4**		

**Coverage**	**MOPHY**	**Random**	***p*-value**	**MOPHY**	**Random**	***p*-value**	**MOPHY**	**Random**	***p*-value**

20%	2.4**	11.6	0.0048	2.8**	10.8	0.0088	4.4*	10.8	0.0121

40%	2.8**	12.0	0.0036	2.8**	10.8	0.0032	4.4*	10.4	0.0179

60%	1.6**	10.8	0.0062	2.4**	11.2	0.0029	3.6**	10.0	0.0054

Performance of MOPHY in capturing the topology of underlying phylogeny for simulated networks. For each parameter setting, the symmetric distance between the underlying tree and the tree reconstructed by MOPHY/randomized method is shown. Reported values are averages over five runs. *p*-values indicate the statistical significance of the performance difference between MOPHY and the randomized method. **: *p *< 0.01, *: *p *< 0.05.

**Table 3:** Performance of MOPHY in capturing the underlying evolutionary distances for simulated networks.

***Most Specific Modules***									
	**Diameter**								

	**2**			**3**			**4**		

**Coverage**	**MOPHY**	**Random**	***p*-value**	**MOPHY**	**Random**	***p*-value**	**MOPHY**	**Random**	***p*-value**

20%	6.87**	16.40	0.0020	6.84**	15.85	0.0017	6.97**	15.78	0.0019

40%	6.81**	16.14	0.0017	6.86**	15.85	0.0017	7.01**	15.53	0.0029

60%	6.79**	15.85	0.0017	6.86**	15.41	0.0016	7.02**	15.25	0.0026

***Most Comprehensive Modules***									

	**Diameter**								
	
	**2**			**3**			**4**		

**Coverage**	**MOPHY**	**Random**	***p*-value**	**MOPHY**	**Random**	***p*-value**	**MOPHY**	**Random**	***p*-value**

20%	8.89	11.72	0.2283	9.67	11.76	0.3512	10.83	11.82	0.6277

40%	7.62*	13.12	0.0277	8.44	11.61	0.2029	9.63	11.29	0.4922

60%	6.70**	14.93	0.0018	7.92	12.90	0.0529	8.96	11.51	0.3263

For each parameter setting, the nodal distance between the underlying tree and the tree reconstructed by MOPHY/randomized method is shown.

**Figure 2:**
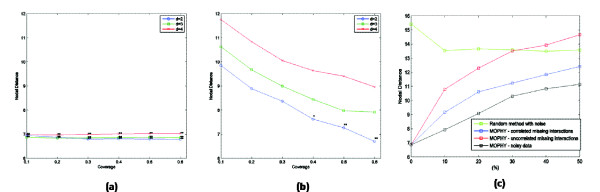
**Effect of Coverage and Noise on Performance**. (a), (b): Performance of MOPHY in capturing the underlying evolutionary distances between simulated networks with respect to coverage (fraction of modules that are used in phylogeny reconstruction). (a) Most specific modules, (b) most comprehensive modules. (c): The effect of noise and missing interactions on the performance of MOPHY. Even when the data is perturbed with 50% noise, MOPHY's accuracy in reconstructing the phylogeny is statistically significant. If the missing interactions are correlated across species, then the effect of missing data is comparable to that of random noise. On the other hand, if they are uncorrelated, then the performance of MOPHY is degraded more quickly, which is expected since the networks break apart in different ways in different species.

**Figure 3:**
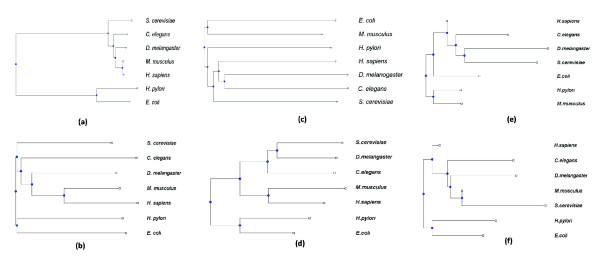
**Comparison of the performances of MOPHY and other methods in reconstructing the phylogenetic tree of seven PPI networks**. (a) Tree based on genome sequences [36], (b) tree reconstructed by MOPHY, (c) tree reconstructed by RDL [16], (d) tree reconstucted by using protein similarities only, (e) tree reconstructed by using random group of proteins as modules, (f) tree reconstructed by Random Homolog Selection method.

• Modularity based analysis is quite robust to noise and missing data in terms of capturing evolutionary relationships, and therefore may be more promising in comparative analysis of extant protein-protein interaction networks, which are highly incomplete and susceptible to noise (Figure 2(c)).

• Network modules are likely to be conserved more in evolutionarily closer species, in terms of the network proximity between the homologs of their constituent proteins (Figure 3).

These results motivate elaborate studies of modular evolution, including identification of module families and reconstruction of evolutionary trajectories for these module families, which in turn will be useful in constructing the "periodic table of systems biology" [6].

## Results and Discussion

Here, we first present all the algorithmic details of our method in *Algorithms *subsection. It is followed by the *Testing *subsection in which results when the algorithm is applied to simulated and real network data are illustrated in a detailed way.

### Algorithms

Our modularity-driven approach to phylogenetic network analysis, MOPHY, which is illustrated in Figure 1, can be summarized as follows:

1. Considering each extant network individually, identify network modules that represent functional and topological properties of each network.

2. Project modules identified on each network to other extant networks, based on the conservation of functional and topological properties, to obtain a *module map *for each species. A module map can be thought of as a mathematical representation of the conservation of extant network modules in the corresponding species.

3. Using module maps, compare networks of diverse species to construct network phylogenies.

4. Using resulting network phylogenies, investigate the evolutionary histories of extant network modules to gain insights on the evolution of functional modularity.

In the rest of this section, we formulate this approach based on biologically sound abstractions and present algorithms to effectively solve the resulting computational problems.

#### Problem Formulation

An interaction network *G *= (*V*, *E*) is specified by a set *V *of proteins and a set *E *of interactions between these proteins. For *v*_*i*_, *v*_*j *_∈ *V*, *v*_*i*_*v *_*j *_∈ *E *indicates that proteins *v*_*i *_and *v*_*j *_interact with each other. Interactions are also associated with reliability scores, specified by weight function *w*: *V *× *V *→ [0, 1], where larger *w*(*v*_*i*_, *v*_*j*_) indicates higher confidence in the existence of an interaction between *v*_*i *_and *v*_*j *_[22].

In this paper, we particularly focus on protein-protein interaction (PPI) networks. However, the proposed method can be easily extended to different sources of data that relates to the organization of cellular systems (*e.g*., gene expression, regulatory networks, metabolic pathways) - to provide an integrated view and analysis of cellular organization. Consider a set  = {*G*_1_, *G*_2_, ..., *G*_*K *_} of *K *PPI networks, each belonging a different extant species.

The proteins in these networks can be associated with each other through a sequence based similarity measure, *ψ*:  ×  → [0, 1], where  = *V*_1 _∪ *V*_2 _∪ ...∪ *V*_*K *_is the set of all proteins in all networks. In practice, we estimate the sequence similarity scores using BLAST *E*-values [1]. Let *E*_*ij *_denote the *E*-value of the most significant bidirectional BLAST hit between proteins *v*_*i *_and *v*_*j *_. We define *ψ *(*v*_*i*_, *v*_*j*_) = 1 + 1/log(*E*_*ij*_), if *E*_*ij *_<*ω*, and 0 otherwise, where *ω *denotes the threshold for the *E*-value being considered significant. If *ψ *(*v*_*i*_, *v*_*j*_) *> *0, *i.e*., *E*_*ij *_<*ω*, we call *v*_*i *_and *v*_*j *_homologs. Note that *ψ *is quite sparse in practice, *i.e*., most proteins have only a few homologs in other species.

#### Network Modularity

An important class of network components that are used in characterizing the networks consists of functional modules [23]. A functional module is in general defined as a set of macromolecules (*e.g*., proteins) that perform a distinct function together (*e.g*., protein complexes, signaling pathways) [5]. It is shown that these functional modules generally manifest themselves in interaction networks as subgraphs with distinctive local properties. For instance, protein complexes would have high inner connectivity in the network, while being somewhat loosely connected to the rest of the network [24]. Furthermore, it is observed that functionally modular groups of proteins and their interactions are likely to be conserved together [9,10,25]. Consequently, modular subgraphs provide an excellent substrate for understanding the evolutionary relationship between networks that belong to diverse species. For this reason, we use conservation of modular subgraphs as an indicator of evolutionary proximity between networks. Note that, comparative methods that target identification of conserved functional modules on a group of networks are also available. However, there are several advantages to our approach of discovering modular subgraphs on each network individually, followed by projection of these subgraphs on other networks: (*i*) multiple graph alignment is a computationally expensive problem [8,15], (*ii*) noisy and incomplete nature of available interaction data makes it particularly difficult to account for mismatches (*e.g*., does a missing interaction indicate divergence of function or is it just an artifact of missing data?), and (*iii*) by identifying modular subgraphs in each extant network individually, we can quantify not only the conservation, but also divergence of modularity in different evolutionary lineages and understand the evolutionary histories of functional modules in diverse species.

##### Proximity as an indicator of modularity

In order to assess the modularity of a group of proteins in a network, we use a proximity based measure, motivated by three key observations: (i) Network proximity is shown to be correlated with functional similarity [26,27]. (*ii*) PPI networks are generally incomplete and prone to noise, therefore existence of alternate paths may account for missing interactions [19]. (*iii*) Existing evidence suggests that existence of alternate paths relaxes the evolutionary pressure on conserving the interaction between two proteins [28].

Consequently, while assessing the modularity of a subgraph in a different network, it might be evolutionarily more relevant to consider the conservation of network proximity rather than conservation of individual interactions.

Let  denote a simple path connecting proteins *v*_*i *_and *v*_*j *_in network *G*. We define the reliability of this path as the aggregate reliability of all interactions on the path, *i.e*., . Then, we estimate the proximity between two proteins with respect to *G *as the reliability of the most reliable path between these two proteins, *i.e*., , where ∏ (*v*_*i*_, *v*_*j*_) denotes the set of all simple paths between *v*_*i *_and *v*_*j *_. Note that, according to this measure, proximity decays exponentially by path length. This is in agreement with the relation between functional similarity and network proximity [26]. If the interactions are not associated with reliability scores (*i.e*., the edges are unweighted, as in many of the existing PPI databases), we assign a score of 1/2 for all interactions, to ensure exponential decay of the proximity measure. In addition, mutual clustering coefficient, as a measure of (sub)graph connectivity, is used to complement proximity in identifying network modules, which is very effective in breaking ties in the unweighted case (please see Section *Module Identification *for details).

##### Projection of proximity on other networks

Now consider evaluating the proximity of two proteins with respect to another network, *G' *≠ *G*, with a view to assess the conservation of their functional association. In order to measure the proximity of *v*_*i *_and *v*_*j *_with respect to *G'*, we aggregate the proximities of their homologs in *V *(*G'*). For this purpose, we normalize the similarity measure with respect to network *G ' *to obtain , where *v*_*i *_∉ *V *(*G'*) and *v*_*k *_∈ *V*(*G'*) Then, the proximity of *v*_*i *_and *v*_*j *_∉ *G' *with respect to *G' *is defined as

(1)

##### Modularity and projection of modularity

We can now define the modularity of a set of proteins with respect to a network. For a set *S *of proteins, all in the same species, *i.e*., *S *⊆ *V *(*G*) for some *G *∈ , the modularity of *S *with respect to network *G*' ∈  is given by

(2)

which is the average proximity between all pairs of proteins in *S *with respect to *G' *In other words, the modularity of a group of proteins in one network with respect to another network measures the extent to which the modularity of the homologs of these proteins is conserved in the corresponding network, through aggregation of proximities of all homologs. Observe that our framework provides a universal view of modularity by facilitating assessment of the modularity of a set of proteins in one species with respect to a network that belongs to another species.

Concerns could be raised that the proposed measure might be heavily influenced by the sequence information, resulting in an inadvertently sequence based analysis (as opposed to network based). However, as we demonstrate in Section *Testing*, the biological signals captured by this measure depend on network topology and the use of sequence information is minimal. Namely, we randomly rewire all interactions (*i.e*., add 100% noise to all networks) and keep sequence similarities as they are. In this case, the performance of the proposed measure in phylogeny reconstruction becomes equivalent to that of a random algorithm (which is otherwise significantly better, even at the presence of 50% noise). This result indicates that biological signal is lost if the network is completely noisy, even if the sequence information is completely preserved.

#### Module Identification

In our framework, network modules are identified on each network individually, and then projected on other networks to construct module maps. In order to identify modular subgraphs in a single network, we use a hierarchical clustering algorithm with a hybrid similarity measure that integrates the concepts of proximity and connectivity for network clustering. Integration of these two alternate measures enables discovery of tightly coupled subgraphs with low diameter.

##### Module identification via hierarchical clustering

We first describe our algorithm based on proximity. For a given network *G *= (*V*, *E*), this algorithm starts with *|V | *distinct clusters, each containing a single protein. Then, it iteratively merges two clusters that are of maximum proximity to each other, until a single cluster containing all proteins in the network is formed. Observe that, this algorithm requires generalization of proximity measures to pairs of clusters. Measures that are commonly used for this purpose are single linkage (for clusters *S*_*i *_and *S*_*j*_, ), complete linkage , and average linkage . We use complete linkage clustering to ensure that all proteins in identified subgraphs are tightly coupled to each other.

##### Proximity vs. connectivity

An important problem associated with the application of proximity based clustering to molecular interaction networks is that, these networks are "ultra-small", *i.e*., distances between most pairs of proteins are very low. Particularly when the interactions are not associated with reliability scores, there are many pairs of proteins with identical proximity at early steps of hierarchical clustering, resulting in many candidate pairs of clusters to be merged. This problem is often alleviated by running the algorithm multiple times with random decisions, and then reconciling the clusters based on the number of times each pair of proteins are assigned to the same cluster.

##### Mutual clustering coefficient complements proximity

In this study, we propose an alternate approach, which uses mutual clustering coefficient [29] between two sets of proteins as a secondary measure of similarity between clusters. Mutual clustering coefficient captures the overlap between the interaction profiles of two proteins, providing an estimate of the likelihood that the two proteins together belong to a functional module. We generalize the notion of mutual clustering coefficient to pairs of clusters (sets of proteins) as follows. For a set *S*_*i *_⊆ *V *of proteins in network *G *= (*V*, *E*), let *N*_*i *_= {*v*_*k *_∈ *V*: ∃ *v*_*l *_∈ *S*_*i *_such that *v*_*k*_*v*_*l *_∈ *E*} be the set of interacting partners of the proteins in *S*_*i*_. Then the mutual clustering coefficient of clusters *S*_*i *_and *S*_*j *_is given by

(3)

Note that this is a measure of the statistical significance of the number of shared interacting partners, which is modeled as a hypergeometric random variable.

Our algorithm uses mutual clustering coefficient as a tie-breaker. In other words, at each step, it merges two clusters with maximum proximity if this pair of clusters is unique. Otherwise, among all pairs of clusters with maximum proximity, it merges the pair with largest mutual clustering coefficient. Consequently, at early stages of the algorithm, mutual clustering coefficient effectively acts as the primary similarity criterion. On the other hand, the proximity criterion ensures formation of clusters with the minimal diameter throughout the course of the algorithm. Once a hierarchical clustering is obtained using this method, we identify the modular subgraphs by properly choosing thresholds on proximity. In Section *Testing*, we evaluate the effect of the proximity threshold on the performance of our algorithms in detail.

#### Constructing Module Maps

Once modular subgraphs are identified on each network, we project these subgraphs to all networks and construct a module map for each network. Module maps can be thought of as feature vectors, where features represent the modularity of each subgraph in the corresponding network. The modularity score of each module with respect to each network is calculated as in Equation 2. To be more precise, assume that we identify *m*_*j *_modules in network *G*_*j *_. Let the set of these modules be . Then, for each network *G*_*i*_, 1 ≤ *i *≤ *K*, the module map *f*_*ij *_of *G*_*i *_with respect to *G*_*j *_is an *m*_*j*_-dimensional vector, defined as

(4)

Consequently, the module map of network *G*_*i*_, 1 ≤ *i *≤ *K *is represented as *f*_*i *_= [*f*_*i*1_, *f*_*i*2_, ..., *f*_*iK*_]. Network modules represent a particular functional component of each network. These modules, when considered altogether, provide a high level representation of each network. In other words, the module maps are signatures of each network and can be utilized to identify the overall phylogeny of these networks.

#### Phylogenetic Tree Reconstruction

Once modules are projected to each network and module maps are created, we use these module maps as feature vectors that characterize the cellular organization in each species. Namely, we compute the pairwise distance between each pair of species by comparing module maps, and apply a traditional phylogenetic tree reconstruction algorithm (*i.e*., neighbor joining [30]) based on these pairwise distances.

Clearly, a straightforward way of estimating evolutionary distances between pairs of species is to consider the correlation between their module maps, *i.e*., to define . However, this method is likely to be significantly affected by the incompleteness of data, since some modules may not exist in some species just because of the unavailability of interaction data. For this reason, while computing the evolutionary distance between two species, we only consider the modules that are identified on the networks of these two species. This avoids the bias introduced by the large number of modules in species for which more comprehensive data is available. Furthermore, we consider one-directional conservation of a single module as a bi-directional hit, to account for missing data. For instance, in the PPI data obtained from DIP, *M. musculus *(mouse) PPI data is relatively incomplete compared to *H. sapiens *(human) PPI data. However, available mouse network is quite similar to parts of the human network. Therefore, almost all modules identified in mouse PPI network are also conserved in human PPI network.

Consequently, by considering only the conservation of modules in the smaller network, *i.e*., defining

(5)

we avoid the effect of missing interactions in the network when incomplete data is used.

#### Simulation of Network Evolution

In order to evaluate and calibrate our algorithms for network based phylogeny reconstruction, we apply our method to synthetic networks that are generated by simulating network evolution. The goal of this simulation is to generate a set of networks with known underlying phylogeny, construct a phylogenetic tree for these networks using our methods, and compare the underlying and reconstructed trees to evaluate the accuracy of our algorithms.

##### Network Evolution Models

We generate synthetic networks by utilizing a model that can accurately reflect the basics of network evolution. There are a variety of duplication based evolution models proposed to model the growth of PPI networks [21,31,32]. These models are inspired by theoretical models of molecular and functional evolution and aim at reproducing the topological properties observed on extant PPI networks. Among these models, we employ a slightly modified version of an iterative model called *duplication- mutation-complementation *(DMC), which is shown to regenerate the subgraph distribution of *D. melanogaster *PPI network significantly better than other network generation models [20]. The DMC model works in iterations and each iteration consists of three steps. (1) *Gene duplication*: A protein *v*_*o *_is chosen uniformly at random and duplicated to create a new protein *v*, with all interactions of *v*_*o*_. (2) *Mutation/Complementation*: For each pair of interactions *v*_*o *_*v' *and *vv'*, one is chosen and deleted with a probability *p*, based on the notion that the duplication results in redundant protein functionality, relaxing the evolutionary pressure on preserving one of the redundant interactions. (3) *Self-Interaction*: An interaction is added between *v*_*o *_and *v *with probability *p'*. In our experiments, we choose *p *= 0.7 and *p' *= 0.1 as suggested in [20].

##### Incorporating Speciation

Unlike other network generation models that focus on evolving a single network, in this work, we evolve multiple networks in parallel to construct a phylogenetic tree of networks. Starting with a single network, the networks evolve concurrently with respect to an evolutionary timeline. We assume that, at each branch, speciation occurs with a constant rate *r*_*s*_, resulting in two networks and splitting the evolution process into two new branches.

Throughout network evolution, a species *S *= (*G*, *n*_*c*_, *n*_*f*_) is specified by its PPI network *G*, the current size of its network *n*_*c*_, and the target size *n*_*f *_. The steps taken while generating the phylogenetic tree of these species can be summarized as follows: Initially, the phylogenetic tree has only one species at the root position with (*G, n*_*c*_, *n*_*f*_), where *G *contains only two interacting proteins, *i.e*., *n*_*c *_is set to 2. The value of *n*_*f *_is assigned randomly from a Gaussian distribution (in our experiments, we use *μ *= 3000, *σ *= 1000 to generate diverse networks with size that reflect that of extant networks accurately).

At each iteration *t *of the evolutionary process, for each species *S *that has not reached to the target interaction network size, *i.e*., *n*_*c*_*< n*_*f*_, the following actions are performed:

• *S *is evolved by a single iteration of the DMC model. This results in the addition of a new node to *G*, incrementing *n*_*c *_by one.

• If the network size reaches the target value after this addition, *i.e*., *n*_*c *_= *n*_*f*_, the network is recorded as a leaf network.

• Otherwise, a speciation event takes place for *S *with probability *r*_*s*_. This process is analogous to generating two new species and replacing the original *S *with an internal node. These new species initially have the same set of interactions with their parent. They are also assigned new *n*_*f *_values right after the speciation, chosen from the same distribution. Note that, common proteins in these new species are recorded as homologs.

When all species reach their targeted network sizes, the simulation terminates with leaves representing the resulting species. Note that each speciation event increases the total number of species by one. The desired number of networks for a given distribution of target network size (number of proteins in each network) can be achieved by properly adjusting *r*_*s *_and the number of iterations.

The evolutionary distances between species are recorded according to the occurrence of speciation events with respect to evolutionary timeline. This enables evaluation of our algorithms in terms of its accuracy in estimating evolutionary distances, as well as the topology of the resulting phylogeny. Furthermore, to obtain similarity scores between proteins in different species, we record homologs after each speciation event and assign scores to each pair of proteins based on the number of duplications that occur after their common ancestor is split into two different proteins. More precisely, we set *ψ *(*P*_*i*_, *P*_*j*_) = 1/*D*_*ij*_, where *D*_*ij *_is the number of duplication events that occur after the common ancestor of these two proteins is split.

### Testing

In this subsection, we evaluate the performance of MOPHY on simulated, as well as real data - in terms of (*i*) success in accurately reconstructing the underlying phylogeny, (*ii*) robustness to noise and missing data, and (*iii*) performance as compared to existing algorithms.

#### Results on Simulated Data

We test our method on synthetic networks generated by simulation of the evolutionary process. In our experiments, in order to keep the size of the networks at a realistic scale with sufficient variability, we set the average number of proteins in a network to 3000, with a standard deviation of *σ *= 1000. Here, average network size is kept relatively smaller as compared to that of extant networks for feasibility constraints, since these experiments are performed multiple times to assess statistical significance and the effect of varying parameters. Our results on extant networks show that the method also scales to larger networks and is applicable in practice. Using this configuration, we generate ten networks for each experiment. For all experiments, we generate five different instances, and for each performance figure, we report the average over these five instances. Note that, in these experiments, the interactions are not associated with reliability scores.

##### Evaluating performance:Comparison of phylogenetic trees

In order to quantify the performance of a tree reconstruction method, it is necessary to compare the reconstructed tree with the underlying tree based on a sound measure of similarity between two phylogenetic trees. For this purpose, we investigate the similarity between the two phylogenetic trees by using two methods: (*i*) Symmetric Difference of Robinson and Foulds [33] is defined as the total number of partitions that are on one tree and not on the other. We use this measure as a metric of success in reconstructing the topology of the phylogenetic tree. (*ii*) Nodal distance [34] takes into account the branch lengths and computes a similarity metric by comparing the sum of distances of every node pair in each tree. We use this method to evaluate the performance of algorithms in capturing the evolutionary distances between different networks.

##### Comparison of performance with other methods

We compare the performance of MOPHY in reconstructing the correct phylogeny to that of four alternate methods

• **RDL**: An existing method for network-based phylogeny reconstruction, which uses relative description length to assess the similarity between networks [16].

• **Random Modules**: This method implements an algorithm similar to that of MOPHY, but it uses random groups of proteins as modules. These random modules are selected in a way that they reflect the modules incorporated by MOPHY in terms of their quantity and size distribution. We use this method as a reference method to assess the contribution of the information on modularity in reconstructing the correct phylogeny.

• **Only Protein Similarity**: This method incorporates only the similarities between proteins to reconstruct a phylogenetic tree. Namely, we still compute feature vectors for each network, but each entry of the feature vector represents the conservation (score of best sequence similarity match) of a single protein. The purpose of using this method as a reference is to assess the contribution of the use of network information (proximity and modularity) in reconstructing the correct phylogeny.

• **Random Homolog Selection**: In this method, we investigate the impact of the assessment of homology on the performance of MOPHY. Namely, homologous proteins across different species are chosen randomly instead of using sequence similarity of proteins. By the comparison of MOPHY against this method, we aim to verify that the assessment of conservation in MOPHY is not arbitrary; MOPHY rather makes effective use of sequence homology to assess conservation of network modules.

The comparison of the performances of these methods over five different instances, obtained through simulation of network evolution, is shown in Table 1. As seen on the table, MOPHY performs drastically better than any of the four alternate methods in terms of minimizing the nodal distance between the correct evolutionary history and the reconstructed evolutionary history. Furthermore, the Random Modules method performs clearly better than RDL, suggesting that incorporation of network proximity, i.e., aggregation of interactions, is more useful than incorporation of network topology, i.e., incorporation of single interactions, in capturing the similarity of networks. However, comparison of the performances of Random Modules and Only Protein Similarity suggests that, when modularity is not considered, incorporation of network information provides marginal improvement. Results obtained by using Random Homolog Selection method are also significantly less accurate as compared to those obtained by MOPHY, indicating that MOPHY makes use of homology information provided by sequence similarity effectively.

##### Design parameters and module selection

In MOPHY, the module identification process can be tuned by adjusting several parameters: (*i*) The threshold on proximity adjusts the trade-off between the tightness and comprehensiveness of modules (higher threshold on proximity results in smaller and more tightly coupled modules). Since the interactions in the simulated networks are unweighted, we use *diameter*, *i.e*., the maximum distance between two proteins in a module, to represent the proximity threshold. (*ii*) As multiple modules are identified in each network, using all modules in phylogeny reconstruction may lead to problems associated with high-dimensionality. Therefore, we investigate the effect of network coverage provided by the modules considered, where *coverage *is defined as the percentage of proteins included in the selected modules. (*iii*) In order to understand which modules are more informative, we consider two different module selection strategies: *most specific*, *i.e*., the set of smallest (with size ≥ 3) modules for a given coverage or *most comprehensive*, *i.e*., the set of largest modules for a given coverage.

##### Performance of MOPHY for different parameters

Detailed statistics on the comparison of underlying and reconstructed phylogenetic trees for systematic experiments on simulated instances are shown in Tables 2, 3, and in Figure 2. To evaluate the performance of MOPHY statistically, we evaluate the statistical significance of its performance with respect to the Random Modules method. The performance difference between MOPHY and Random Modules can be thought of as an indicator of the usefulness of relying on conservation of modular network structures as opposed to arbitrary (groups of) proteins and their interactions. We quantify the statistical significance of the performance difference between MOPHY and Random Modules based on Student's *t*-test to compare the means of two populations. The *p*-value for an experiment gives the probability that an algorithm that incorporates sequence conservation and network proximity, but not modularity can achieve as good as MOPHY solely based on chance. As seen in Table 2, for any configuration of parameters, the accuracy of the topology of the phylogenetic tree reconstructed by MOPHY is highly significant. In general, more specific (smaller) modules appear to be more informative. Indeed, as seen in Table 3, when evolutionary distances are considered, the performance with more comprehensive (larger) modules is not statistically significant. Furthermore, performance degrades with increasing diameter (less proximity), suggesting that conservation of tightly coupled modules is more informative in reconstructing evolutionary histories. The effect of coverage on performance is shown in Figure 2(a) and 2(b). When more specific modules are used, the effect of coverage on performance is marginal. This indicates that careful selection of a concise set of small, tightly coupled modules may be adequate to reconstruct network phylogenies accurately. Finally, it is interesting to note that the randomized method performs better with large clusters, which is probably due to the increased likelihood that a random group of proteins will contain an informative subset of proteins.

##### Robustness against noise and missing data

Currently available PPI data is likely to be highly noisy and incomplete. Hence, we evaluate the robustness of MOPHY against random noise and incompleteness of data. For the purpose of observing the effect of noise, after generating the networks via simulation of network evolution, we randomly perturb the resulting networks by repeatedly swapping randomly selected interactions. Furthermore, in order to evaluate the effect of missing interactions in our experiments, we apply two interaction removal strategies, namely *uncorrelated *and *correlated *removals. For the *uncorrelated *removal method, a certain percentage of protein interactions in each separate network is removed randomly. Whereas for the latter removal method, if an interaction is selected for removal from one network, then one of the interactions among the homologs of the interacting proteins is also removed from all the other networks where it exists.

The behavior of the performance of MOPHY with respect to noise rate (percentage of interactions that are swapped) and missing interactions is shown in Figure 2(c). These experiments are performed for *diameter *= 3, *coverage *= 60%. As seen in the figure, although the accuracy of MOPHY decreases with noise and missing interactions as expected, the performance difference between MOPHY and the randomized method is significant even at the presence of 50% noise or 40% correlated missing data.

This observation suggests that MOPHY can be used to extract meaningful information on evolutionary histories of networks even when the networks are highly noisy and incomplete. On the other hand, the performance of MOPHY degrades more rapidly with increasing number of uncorrelated missing interactions. This is expected since in the case of uncorrelated missing interactions, after a sufficient number of interactions are removed, the network distance between the homologs of two interacting proteins in one species becomes infinite in the network of another species.

However, as evident in Figure 2(c), MOPHY's performance is significant (*p <*0.01) with respect to the random algorithm even when 20% of the interactions are removed from the networks at random. Moreover, note that the performance of the randomized method is not affected by noise, and the performance of MOPHY becomes equivalent to that of the randomized method at the presence 100% noise (*i.e*., random edge swapping is repeated for a sufficiently large number of iterations). These results indicate that the biological signals captured by MOPHY depend on network topology and the use of network proximity and modularity provide significant information on conservation of function that is beyond sequence similarity.

#### Results on Extant PPI Networks

We test our method on the available PPI networks from seven diverse species. The PPI data is obtained from the Database of Interacting Proteins (DIP) [35]. These networks include those of *D. melanogaster *(7471 proteins, 22656 interactions), *S. cerevisiae *(4968 proteins, 17286 interactions), *E. coli *(1848 proteins, 5930 interactions), *C. elegans *(2646 proteins, 3977 interactions, *H. sapiens *(1334 proteins, 1539 interactions), *H. pylori *(710 proteins, 1359 interactions), and *M. musculus *(414 proteins, 337 interactions). Although the network sizes in this database vary dramatically for different species, MOPHY can effectively deal with such incompleteness by considering each pair of species separately and considering the conservation smaller network's modules in the larger network (as discussed in Section *Phylogenetic Tree Reconstruction*).

To reconstruct the phylogeny of these seven networks via MOPHY, we use the most specific modules that contain at least three proteins and set the coverage to 50%. While identifying homolog proteins, we set the BLAST *E*-value threshold score *ω *to 0.05. As in our experiments on simulated data, we compare MOPHY with four alternate methods; (*i*) RDL, (*ii*) using random modules, (*iii*) using only protein similarities, and (*iv*) random homolog selection method. For reference, we also consider the phylogenetic tree that is reconstructed based on sequenced genomes [36], which is shown in Figure 3(a). The phylogenetic trees reconstructed based on the seven PPI networks by MOPHY, RDL, using only protein similarities, using random modules and random homolog selection method are shown in Figures 3(b), (c), (d), (e)) and 3(f) respectively. Unlike other methods, the tree reconstructed by MOPHY complies well with common knowledge on the underlying phylogeny of these seven diverse species and is also consistent with the whole genome based phylogeny. It should be noted that, as evident in Figure 3, network-based distance measures tend to overestimate evolutionary distances between extant species. Therefore, methods for normalizing the estimated distances between networks are necessary.

Incidentally, these results also provide evidence supporting the *Coelomata *topology in the *Coelomata vs. Ecdysozoa *debate regarding the evolutionary relationship between nematodes, anthropodes, and vertebrates, which has also been supported recently through rigorous analysis of the conservation patterns in intron positions [37]. It is worth to note that, due to limited availability of data, PPI networks differ significantly in size from one species to another. This actually introduces a lot of artificial variation between networks, which might, on a common graph measure, overwhelm desired biological signals. Indeed, as seen in Figure 3(c), RDL is significantly affected by the variability in data availability; it assigns mouse PPI network to the same clade with prokaryotic networks, presumably because the interaction data for this species is quite limited. On the other hand, by focusing on the signals harbored by some more informative modules, we avoid the interference of this global difference among networks. The discriminative power of MOPHY suggests that functional modules identified on PPI networks are important carriers of evolutionary messages. These functional hotspots convey some information beyond that of the apparent graphical variation among the networks, which help overcome the artificial bias commonly introduced to PPI networks by noise or unavailability of protein-protein interactions.

## Conclusion

In this paper, we propose a phylogenetic framework for analyzing modularity in protein-protein interaction networks. Our approach is motivated by the premise that biomolecular interactions and their modularity are likely to provide direct functional information on the evolution of biological systems. We also develop a method based on the simulation of network evolution to evaluate phylogenetic tree reconstruction methods. Comprehensive experimental results on simulated, as well as real data show that our algorithm is highly successful in reconstructing the underlying phylogenies based on PPI networks, is quite robust to noise, and performs significantly better than existing network-based phylogeny reconstruction algorithms on available protein-protein interaction data. These results demonstrate the promise of modularity-based approaches in comparative network analysis and motivate the study of the evolution of network modularity within a phylogenetic framework.

## Authors' contributions

SE and XL contributed equally to the manuscript. All authors contributed in the planning of the study and development of algorithms. SE, XL and GB implemented and tested various parts of the framework. JL and MK coordinated the study and drafted the manuscript. All authors contributed to finalize the manuscript. All authors read and approved the final manuscript.
